# Could ‘*Isochoric Freezing*’ Revolutionise Food Preservation?

**DOI:** 10.3390/foods13111762

**Published:** 2024-06-04

**Authors:** Kostadin Fikiin, Stepan Akterian, Alain Le Bail, James K. Carson, Trygve M. Eikevik

**Affiliations:** 1 Refrigeration Science and Technology, Department of Heating and Refrigeration Engineering, Technical University of Sofia, 1000 Sofia, Bulgaria; 2 Process Engineering for Food and Aromatic Products, University of Food Technologies, 4002 Plovdiv, Bulgaria; s_akterian@uft-plovdiv.bg; 3 ONIRIS—GEPEA UMR CNRS 6144, 44300 Nantes, France; alainlebailpro@gmail.com; 4 School of Engineering, University of Waikato, Hamilton 3240, New Zealand; 5 Department of Energy and Process Engineering, Faculty of Engineering Science and Technology, Norwegian University of Science and Technology, 7034 Trondheim, Norway; trygve.m.eikevik@ntnu.no

**Keywords:** food, refrigeration, cooling, chilling, freezing, isochoric freezing, cold chain, high pressure, energy, sustainability

## Abstract

The present article responds to the food engineering community’s growing interest in an emerging and lauded approach to food preservation, popularised by its developers as ‘*isochoric freezing*’. A strong campaign in the scientific literature and mass media has recently promoted this technique as a universal replacement for traditional food freezing and the frozen supply chain by highlighting a number of alleged advantages of ‘*isochoric freezing*’. Some of these claims therefore require a more neutral and critical assessment against the background of the today’s state of the art in food freezing technologies. Hence, this article spotlights several concerns regarding the plausibility, energy expenditure, resource efficiency, process rate, throughput and safety of ‘*isochoric freezing*’, as well as the correct use of food refrigeration terminology. The aspects considered are intended to make food scientists, technologists and engineers more aware of the real capabilities and the application perspectives of this still immature mode of refrigerated food processing.

## 1. Introduction

A low-temperature preservation technique, launched under the name ‘*isochoric freezing*’ [1] and initially trialled in cryobiological studies, has recently received significant attention after a series of public releases claiming that it can outperform the traditional isobaric freezing of foods throughout the entire cold chain, from production to consumption [2]. The proposed method has been advocated for as a game-changing revolution in the preservation of foods [3,4,5], a ‘*panacea*’ to address most challenges of food storage [6] or a ground-breaking innovation to respond to both the energy- and quality-related demands of the modern frozen food industry worldwide [3,7,8].

**Figure 1:**
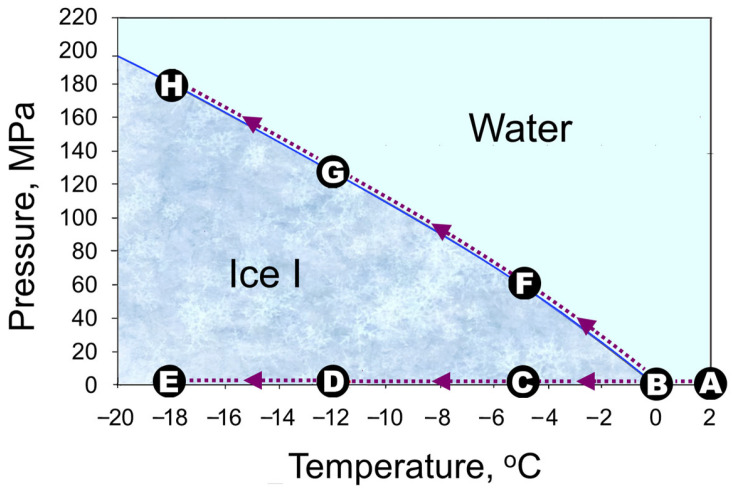
A comparison between constant-pressure (isobaric) freezing at barometric pressure, A-B-C-D-E, and isochoric pressure-aided supercooling (‘*isochoric freezing*’), A-B-F-G-H, down to −5, −12 and −18 °C.

The underlying principle of ‘*isochoric freezing*’ is based on hindering the phase transition of food’s liquid water to ice by applying high pressures ranging from about 25 MPa to 180 MPa, thereby supercooling food to a final temperature of approximately −2 to −18 °C, as depicted on the pressure–temperature chart for water (Figure 1). Such great pressurisation is achieved under constant-volume (isochoric) thermodynamic conditions by decreasing the temperature inside rigid containers holding a sufficient amount of an auxiliary liquid substance (typically an aqueous solution), filled around the food items, which seeks to expand its volume as it freezes [2,7,8,9].

The beneficial influence of ‘*isochoric freezing*’ on food quality is attributed to the reduced structural damage thanks to the inhibited phase change inside the food [9]. With reference to the avoided ice crystallisation with the release of latent heat, which reduces the cooling load, the method’s developers claim enormous potential savings of energy for refrigeration [7,8].

As an expert team, set up within the *International Institute of Refrigeration* (IIR), we have recently examined the current state of play in the field of ‘*isochoric freezing*’, and it is our opinion that a number of significant points require close scrutiny or critical re-evaluation [10,11].

## 2. Terminology

The method’s proponents believe that the phrase ‘*isochoric freezing of food*’ is ‘*the most rigorous thermodynamic descriptor*’ [12], although the process aims to keep food unfrozen. By contrast, our expert group [10] considers the latter incompatible with science-based terminology [13,14] but possibly admissible as a commercial tradename.

Thus, ‘*isochoric pressure-aided supercooling*’ or ‘*isochoric pressurised supercooling*’ is deemed a better title for the technology [9,10,15]. ‘*Isochoric freezing*’ proponents disagree with such a formulation, arguing that the spontaneously supercooled state is metastable [12]. Nevertheless, the established international terminology defines supercooling as ‘*cooling a substance below the normal freezing point without solidification*’ [13,14], which is not necessarily relevant to thermodynamic stability. As is widely known, artificial supercooling for useful purposes can be achieved by using different physical means (pressure, electrical and/or magnetic fields, structural modifications, emulsification, demineralisation, etc.).

In fact, the expression ‘*isochoric freezing of foods*’ does not make physical sense, because freezing occurs, by definition, in the food-surrounding auxiliary substance, while the water contained in the pressurised food items remains basically in the liquid phase [2,7,8].

## 3. Plausibility, Resource Efficiency and Safety

As the enduring preservative effect of freezing is principally due to the conversion of liquid water into ice, rather than to the lowered temperatures, the supposed method’s benefits need to be verified against the classical modes of long-term food storage. To date, no replicable shelf-life data have been obtained for isochorically supercooled food products. For delicate and highly porous foods, the tissue-damaging impact of elevated pressures might be no less severe than the phase-change-caused destruction [9]. Thus, isochoric preservation is a largely unexplored topic, whose capabilities still need to be investigated and hopefully proven in a sufficiently representative real-life environment.

Achieving and maintaining the necessary high pressures along the food cold chain for freezing, frozen storage and distribution involve the use of bulky pressure-withstanding containers [7,8,12,16] possessing large thermal masses (i.e., high heat capacities). This greatly increases the overall usage of energy, constituent materials and labour for the production, refrigeration, storage, transportation and handling at home of such massive containers (given also that the great pressurisation inside them is a safety hazard and a potential source of explosion accidents). Furthermore, the utilised containers and auxiliary substances need to be collected, reused or recycled, thereby involving additional operations and efforts. All of these circumstances make ‘*isochoric freezing*’ more resource-intensive than the traditional (isobaric) freezing at barometric pressure [9].

## 4. Process Rate

A typical isochoric system, consisting of a rigid container, pressurising freezable solution and food item, is much slower to cool down than the same quantity of food undergoing conventional isobaric freezing through close or direct contact with the refrigerating medium. The process throughput is thus limited, while the effects of the low processing rate on the end product’s quality should also be investigated and taken into account. Unlike traditional freezing, where continuous processing is common, the isochoric approach is more feasible in a batch mode of operation.

## 5. Energy Demand and Efficiency

**Figure 2:**
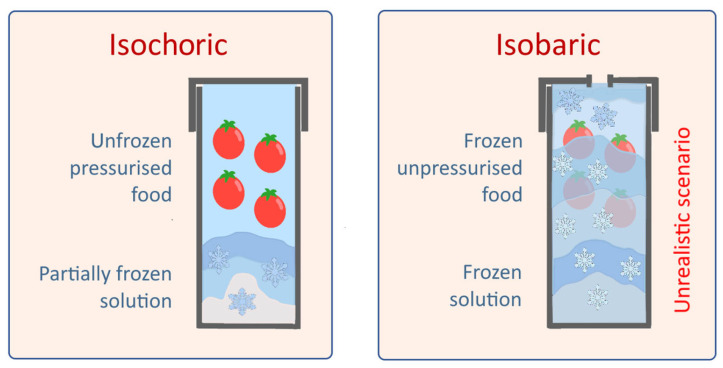
Unrealistic energy-wasting scenario for traditional (isobaric) freezing to be compared with ‘*isochoric freezing*’ (as assumed in [7]).

The enormous energy benefits claimed [3,7,8] are debateable as it remains unclear how and where the energy consumed to cool the high-pressure container and to freeze the pressurising substance is taken into account. In particular, the claim for up to 70% energy savings [7], as compared with the classical isobaric freezing, remains unproven. The method’s proponents [7] claimed these huge savings after comparing the calculated cold energy expenditure of an isochoric system with that of its isobaric counterpart by using the assumption depicted in Figure 2.

However, presenting classical isobaric freezing at atmospheric pressure, as per the right-side image of Figure 2, is misleading and unrealistic, because no technology freezes food in this way. Unlike ‘*isochoric freezing*’, classical freezing requires neither special robust containers nor pressurising substances, because direct or close contact between the product and the refrigerating medium is normally present [10]. Energy comparisons can only be objective under realistic conditions and per unit of useful end product (preserved food), without arbitrarily attributing any imaginary energy wastage to the classical freezing. The alleged ‘*isobaric system*’ (Figure 2) does not truly exist in food freezing practices and cannot serve as a reference.

Whilst no quantified energy usage data have been published so far about the method’s energy consumption for a ‘*workable industrial configuration*’ [12], the energy assessments [9] of experimental trials [16] do not report favourably on the energy performance of ‘*isochoric freezing*’.

## 6. General Observations and Discussion

A number of thermophysical phenomena and application-related issues regarding ‘*isochoric freezing*’ remain to be explored in more depth. It should clearly be pointed out that this technology does not rely on freezing the water contained in the food matter of interest, thereby representing a non-freezing concept. The spectacular reports of its exceptional energy efficiency [7,8] need to be critically re-examined. The energy usage must be determined per unit of saleable end product or useful effect, which is a ‘*conditio sine qua non*’—an irrevocable prerequisite for any credible technology assessment.

Energy might potentially be saved by carrying out the refrigerated warehousing and distribution of pressurised ‘*isochorically pre-frozen*’ products at a higher temperature (e.g., −5 °C instead of the standard −18 °C) [10]. However, such hypothetical savings are unlikely to offset the resource and cost inefficiencies of processing, packaging, transport and retail, given also the safety hazard of high-pressure operations for personnel and consumers [9,10].

As long as the water in the food remains unfrozen in ‘*isochoric freezing*’, the effective control of microbial growth is challenging. While freezing and frozen storage ensure low moisture content and water activity, the liquid phase state of water (both in the food itself and in microbial cells) results in a different microbiota composition with subsequent food safety and quality implications [17]. Moreover, some food macromolecules (e.g., food proteins) tend to aggregate during long-term storage, which affects food quality attributes [18]. When such molecules are not separated by frozen water, the pressure combined with the unfrozen moisture state may promote protein aggregation, thereby compromising the quality and shelf life.

Instead of campaigning for the introduction of ‘*isochoric freezing*’ as a universal replacement for classical freezing, the method might be promoted more reasonably as a relatively simpler alternative to the existing complex technologies for the high-pressure processing of foods by expensive hyperbaric equipment. A single ‘*isochoric freezing*’ application at the processing stage is much more realistic than maintaining the high pressure along the entire cold chain for food preservation, from producer to consumer. As in the other known modes of high-pressure treatment, juices, concentrates and liquid foods are especially appropriate for low-temperature pressurisation under isochoric conditions. A scenario where the liquid product itself freezes isochorically, thus serving as a self-pressurising agent, is a more appealing and less expensive modality than pressurising and supercooling the product by means of an auxiliary freezable liquid substance filled in the isochoric container [19].

## 7. Conclusions

Our IIR expert group concluded that, regardless of the optimistic statements made by proponents of ‘*isochoric freezing*’, at present there is no convincing evidence that the suggested method can compete (in practical or economic terms) with classical (isobaric) food freezing and the frozen supply chain operating at barometric pressure [10]. While judicious technology transfer from cryobiology to the food sector is welcome, any promotional campaign for the mass application of isochoric preservation in the food refrigeration industry should be grounded on sound and incontestable practical results.

## Data Availability

This study analysed published information and data available in the cited sources. Data sharing is not applicable to this article.
